# Surveys of public knowledge and attitudes with regard to antibiotics in Poland: Did the European Antibiotic Awareness Day campaigns change attitudes?

**DOI:** 10.1371/journal.pone.0172146

**Published:** 2017-02-17

**Authors:** Beata Mazińska, Izabela Strużycka, Waleria Hryniewicz

**Affiliations:** 1 Department of Epidemiology and Clinical Microbiology, National Medicines Institute, Warsaw, Poland; 2 Department of Dental Comprehensive Care, Medical University of Warsaw, Warsaw, Poland; Tallinn University of Technology, ESTONIA

## Abstract

**Background:**

Antimicrobial resistance is a global public health problem. Monitoring the level of knowledge regarding antibiotics is a part of the European Union Community strategy against antimicrobial resistance.

**Objective:**

To assess knowledge by the general public in Poland regarding antibiotics, AMR, and the impact of the European Antibiotic Awareness Day campaigns.

**Methods:**

The repeated cross-sectional study was developed and carried out among the general public in Poland (in 5 waves between 2009 and 2011, embracing a total of 5004 respondents). The survey was based on a self-designed questionnaire, and carried out by Millward Brown SMG/KRC, using Computer Assisted Telephone Interviews (CATI).

**Results:**

A high percentage of Polish adults had used antibiotics within the 12 months preceding their participation in the study (38%). Statistically relevant differences were observed regarding the respondents’ gender, age, education and employment status. The majority of the antibiotics used were prescribed by physicians (90%). In all five waves, 3% of the respondents purchased an antibiotic without a prescription. Prescriptions were mostly obtained from a general practitioner. The prevailing reasons for taking antibiotics were the common cold, sore throat, cough and flu. Approximately 40% of the respondents expected a prescription for an antibiotic against the flu. The vast majority knew that antibiotics kill bacteria (80%) but at the same time 60% of respondents believed antibiotics kill viruses. Physicians, pharmacists, hospital staff and nurses were mentioned as the most trustworthy sources of information. A third of the respondents declared to have come across information on the prudent use of antibiotics in the preceding 12 months. In the fifth wave, nearly half of the participants (48%), who had come across information about antibiotics in the preceding 12 months declared that the information resulted in a change in their attitude towards antibiotic use.

**Conclusion:**

The survey generated information about the knowledge, attitude, and behavior regarding antibiotics among the general population of Poland. Inappropriate antibiotic use is still highly prevalent in Poland, although a positive trend in behavioral change was observed after the educational campaigns. Additional didactic and systematic education campaigns regarding appropriate antibiotic use are needed and the use of the Internet as an education tool should be enhanced.

## Introduction

The discovery of antibiotics is considered one of the most important achievements of 20^th^ century medicine. After many years of successful usage antibiotics have begun to lose effectiveness due to growing antimicrobial resistance (AMR) [1].

AMR has been recognized by WHO and the European Commission (EC) as one of the most serious threats to public health. A serious warning was issued in an April 2014 WHO document " Antimicrobial Resistance: Global Report on surveillance". It indicated that the "post-antibiotic era, in which common infections and minor injuries can kill, is far from being an apocalyptic fantasy but instead a very real possibility for the 21^st^ century" [2].

The major problem lies in the imprudent use or overuse of antibiotics in human medicine and animal husbandry as well as a lack of awareness of the scale of the problem and sequelae of antimicrobial resistance [3]. One proposed immediate solution is the rationalization of antibiotic use [4]. This makes the education of healthcare professionals and the general public vitally important.

The EU has recognized this and emphasizes educational campaigns on AMR for the general public, healthcare, veterinary medicine, and other professionals. Since 2008, the European Centre for Disease Prevention and Control (ECDC) has coordinated November 18th as the “European Antibiotic Awareness Day” (EAAD), a European health initiative that provides a platform and support for national campaigns to raise awareness on the prudent use of antibiotics [5]. Poland has actively joined this initiative; the study presented is aimed at assessing the knowledge of the general public in Poland with regard to antibiotics, AMR, and the impact of the EAAD campaigns.

## Materials and methods

### Study design and population

The cross-sectional study was conducted in Poland in five waves between 2009 and 2011: before (October) and after (December) the EAAD campaigns in 2009 and 2010 and following the campaign in December 2011. Campaign educational materials were prepared following the guidelines given by the ECDC and intensive educational action aimed at the general public was undertaken. Most of the following activities were undertaken every year, since 2008 until the present. They included: press conferences, posters, leaflets for patients, leaflets for children and parents, exhibition displayed nationwide (16 posters), 10 sec. spot broadcast in public TV, 10 sec, 30 sec. spots in public TV, Facebook since 2011.

Adults (aged 18 years and older) were selected from the general population by multistage stratified random sampling. Respondents were divided into five age groups (years): 18–24, 25–34; 35–44, 45–59 and ≥60. In addition, they were grouped according to gender (male, female), education (primary education, trade school, secondary education, university education) and employment status (employed, unemployed, high school/university students, retired). The characteristics of the respondents against the background of the whole population of Poland are summarized in Table 1.

**Table 1:** Characteristics of respondents compared with the Polish general population.

Characteristics	Study, Respondents n = 5004 (%)	Polish population 31 December 2011, 38538* *in thous*. (%)
**Sex**		
Male	2243 (44.8)	18654.6 (48.4)
Female	2761 (55.2)	19883.9 (51.6)
**Age groups**		
18–24	513 (10.3)	3762.2 (12.0)
25–34	887 (17.7)	6381.1 (20.3)
35–44	812 (16.2)	5275.0 (16.8)
45–59	1508 (30.1)	8176.9 (26.0)
≥60	1284 (25.7)	7796.5 (24.8)
**Education**		
Primary not completed	-	354.0 (1.1)
Lower secondary and primary	400 (8.00)	7094.2 (21.7)
Trade school	960 (19.2)	7260.7 (22.2)
Secondary education	2195 (43.9)	9705.6 (29.7)
Post-secondary	-	868.1 (2.7)
University education	1442 (28.8)	5690.2 (17.4)
Refused to answer (unknown)	7 (0.1)	1706.8 (5.2)
**Employment (source of maintenance)**		
Employed	2402 (48.0)	14233 (52.2)**
Unemployed	473 (9.5)	1983 (7.3)
Post secondary/university students	368 (7.4)	2094.7 (7.7)
Retired	1761 (35.2)	8970.7 (32.9)

*As of 31.12.2011 Statistical Yearbook of the Republic of Poland 2012

** Economic activity of the population aged 15 and more. Statistical Yearbook of the Republic of Poland 2012

### The questionnaire

The survey was developed by the first author and partly based on the “Antimicrobial Resistance” Eurobarometer Survey 338 with additional questions regarding visibility of the problem in our country and impact of the campaign.

The questionnaire was consulted with specialists from different fields (sociologists, psychologists, public health specialists, health promotion specialists) with regard to its logic and clarity of content.

The questionnaire was divided into two parts. The first part of the questionnaire investigated the socio-demographic characteristics of the respondents. The second part of the questionnaire was divided into five sections and the following questions were addressed:

1. Use of antibiotics, including previous antibiotic exposure
2. Knowledge about antibiotics
3. Attitudes and behaviors regarding antibiotics
4. Sources of information
5. Knowledge and impact of the campaign on attitudes towards the use of antibiotics.

Detailed topics of the questionnaire are attached at the end of this paper (appendix).

### Data collection

The survey was carried out by Millward Brown SMG/KRC, using Computer Assisted Telephone Interviews (CATI).

The survey performed in the study was of the Omnibus type. For Omnibus type surveys in Poland, N = 1000 was a standard sample. In N = 1000 sample the maximum measurement error is +/- 3,2%. When 50 respondents were added the error declines only marginally, therefore it is not cost-effective to increase the sample. The survey sample of 1000 respondents was a random representation of the Polish adult population.

The sampling frame for the survey was a telephone number database created by MB SMG/KRC using available 5-digit prefixes by inserting all possible 2-digit suffixes to each prefix. The database assigned a telephone number to the administrative unit. It was a list assisted random digit dialing method. It provided access to practically all households equipped with a landline, regardless of the fact whether the number has ever been listed in telephone directories.

Company telephone numbers, deactivated telephone numbers as well as MPS numbers were indicated in the database. The survey respondents were chosen at random within the selected strata. Stratification included the size of locations (9 divisions), their placement within new voivodships (16) as well as the respondents' age and sex. The 2010 stratification was based on demographic data published by Central Statistical Office (http://stat.gov.pl/) on June 30, 2009 (most recent).

The sample was created in stages. The first stage included the stratification of the Polish population with reference to their location (within 16 voivodships) and the size of the location. The sample included towns as well as villages.

Locations are assigned within the chosen strata. Those locations would later form randomization units. The sample included all towns with population of over 50000 inhabitants. In each of these self-representative towns a simple random sample was chosen from all telephone numbers available in the database. For strata including locations with smaller populations (towns as well as villages), a simple random sample was chosen from all telephone numbers available in each voivodship. Different strata regarding villages, cities with population below 10000 / 20000 / 50000 inhabitants were distinguished. The sample sizes of the particular strata (both self-representative towns and locations from various size divisions within voivodships) were proportionate to the number of inhabitants in the whole population of the country.

Due to continuing decreasing of the number of landlines in households and because of improved availability of respondents (some of them, especially young, are not available via landlines), an additional 50% of the sample was chosen from mobile phone numbers. The mobile sample was chosen similarly to the landline sample (according to prefixes from http://www.urtip.gov.pl) provided that information about the location was verified at the beginning of the interview. The computer was responsible for appropriate sample stratification.

At the stage of phone number randomization a 5-20x multiplication of the sample might occur due to the possibility of selecting a phone number that is either non-existent, temporarily unavailable, fax-connected or owned by a company. The above additive also includes cases when: the subscriber cannot be reached, the line is engaged, the respondent refuses to take part in the survey, the respondent does not meet the criteria (e.g. age).

Regardless of the telephone number randomization, groups of respondents from different strata were secondarily stratified according to two additional criteria: sex and age. There were 5 age divisions (18–24, 25–34, 35–44, 45–59 and 60–75 years). The obtained numbers were the basis for defining the sample size in different strata. The final stage of selection for the trial for the survey took place during the phone interview. The computer system monitoring the survey collected data regarding chosen telephone numbers as well as predicted number of different strata (for macro regions, locations of different size, gender and age groups). The computer provides interviewers with telephone numbers at random. In the initial phase of the interview the interviewer chose a respondent using a standard method and the system automatically monitored the maintenance of the appropriate stratification.

### Ethics

In all cases verbal consent was always obtained from the respondents at the beginning of the interview. Participation in the survey was voluntary. At the beginning of each interview, the researchers introduced themselves, stated where they were calling from and asked whether the respondent agrees to be interviewed.

Polish telecommunication law does not forbid calling random phone numbers, provided that the calls are not telesales and the respondent agrees or refuses during the arrangement, a written consent is not required. The interviews were recorded and stored for 3 months.

The approval of the Ethics Committee is required in Poland only if sensitive personal data are used or if the study involves the administration of an active substance or therapy.

However this study was approved by the Ethics Committee of Warsaw Medical University (Registration number: AKBE/45/13).

### Data analysis

Data were analyzed statistically with the IBM SPSS Statistics for Windows 19.0. software package. Some variables were recoded to chosen categories including binary coding to be used in logistic regression. To identify the differences in distribution of general knowledge about effectiveness and using antibiotics between the study group (eg. sex, or waves of survey) chi-square tests were performed. Furthermore, to identify the sociodemographic factors related to general knowledge about the effectiveness and use of antibiotics multiple logistic regression was used. The reference groups were set as age 18–24, education—higher degree, and employed. Odds ratios (ORs) with corresponding 95% confidence intervals (CIs) were calculated. For all test P-values of 0.05 or less were considered to be statistically significant.

## Results

Altogether 5004 respondents were recruited: 1000 in wave 1—(October 2009), 1002 in wave 2—(December 2009), 1001 in wave 3 (October 2010), 1000 in wave 4 (December 2010) and 1001 in wave 5 (December 2011). The response rates were 5.1% -11.3% (2009–2011).

The results presented below indicate an average for the five waves of the study (2009–2011).

### Use of antibiotics (previous antibiotic exposure)

A high percentage of Polish adults had used antibiotics within the previous 12 months (38%). The percentage remained at a similar level throughout the whole study (5 waves). Women (40%) were more likely than men (36%) to have taken antibiotics in the preceding year (OR = 0.84, 95% CI = 0.74–0.94)

The respondents in the group 18–34 years old (43.5%) were more likely to have taken antibiotics than those above 60 years of age (35%) (OR = 0.71, 95%CI = 0.52–0.97). Students were more likely to take antibiotics than those who are employed (OR = 1.63, 95% CI = 1.20–2.12)

As can be seen in Table 2, the prevailing reasons were: common cold (30%), sore throat (23%), “flu” (16%) and cough (15%). There were significant differences between socio-demographic groups concerning the reason for taking antibiotics, particularly in the case of flu. Males were more likely than females to use antibiotics when suffering from flu, 18% and 14% (OR = 1.28, 95% CI = 0.99–1.65), respectively or for common cold, 34% and 27%, respectively (OR 1.34, 95% CI = 1.09–1.64). The respondents most likely to take an antibiotic for the flu were between ages 18 and 24 (26%). Older people rarely declared taking antibiotics because of the flu (OR = 0.33, 95% CI = 0.18–0.62). People with primary education (OR = 1.94, 95% CI = 1.18–3.21) and trade school (OR = 2.02, 95% CI = 1.37–2.97) took antibiotics because of the flu twice as often as people with higher education.

**Table 2:** Reason for last taking of an antibiotic *(socio-demographic profile)*.

Characteristics	n	Cold n (%)	Adjusted ORs (95% CI)	sig.*	Flu n (%)	Adjusted ORs (95% CI)	sig.	Cough n (%)	Adjusted ORs (95% CI)	sig.	Sore throat n (%)	Adjusted ORs (95% CI)	sig.
**Total**	1919	575 (30)			303 (16)			294 (15)			445 (23)		
**Sex**													
Male	810	275 (34)	1	-	147 (18)	1	-	125 (15)	1	-	172 (21)	1	-
Female	1109	300 (27)	1.34 (1.09-1.64)	**0.005**	156 (14)	1.28 (0.99-1.65)	0.061	169 (15)	0.97 (0.75-1.26)	0.835	273 (25)	0.82 (0.66-1.02)	0.076
**Age groups**													
18-24	236	73 (31)	1	-	62 (26)	1	-	45 (19)	1	-	67 (28)	1	-
25-34	363	120 (33)	1.39 (0.88-2.22)	0.161	63 (17)	0.62 (0.37-1.04)	0.070	58 (19)	0.94 (0.54-1.65)	0.830	84 (23)	0.75 (0.47-1.22)	0.253
35-44	292	88 (30)	1.22 (0.74-2.01)	0.434	49 (17)	0.56 (0.32-0.97)	**0.038**	41 (14)	0.77 (0.42-1.41)	0.395	76 (26)	0.88 (0.53-1.47)	0.630
45-59	583	170 (29)	1.18 (0.74-1.9)	0.483	81 (14)	0.42 (0.25-0.71)	**0.001**	81 (14)	0.73 (0.42-1.29)	0.284	129 (22)	0.72 (0.44-1.17)	0.181
≥60	445	124 (28)	1.2 (0.71-2.05)	0.494	48 (11)	0.33 (0.18-0.62)	**0.001**	69 (16)	0.86 (0.45-1.64)	0.653	89 (20)	0.66 (0.38-1.15)	0.140
**Education**													
University education	547	159 (29)	1	-	65 (12)	1	-	68 (12)	1	-	129 (24)	1	-
Primary education	162	48 (30)	1.14 (0.76-1.72)	0.520	32 (20)	1.94 (1.18-3.21)	**0.009**	30 (19)	1.62 (0.98-2.67)	0.057	40 (25)	1.05 (0.68-1.62)	0.820
Trade school	352	116 (33)	1.27 (0.94-1.72)	0.122	72 (20)	2.02 (1.37-2.97)	**<0.001**	63 (18)	1.67 (1.13-2.47)	**0.010**	85 (24)	1.06 (0.76-1.47)	0.742
Secondary education	854	251 (29)	1.07 (0.83-1.36)	0.613	133 (16)	1.38 (0.99-1.92)	0.061	133 (16)	1.31 (0.95-1.82)	0.102	191 (22)	0.92 (0.71-1.21)	0.562
**Employment**													
Employed	895	278 (31)	1	-	144 (16)	1	-	133 (15)	1	-	209 (23)	1	-
Unemployed	167	46 (28)	0.86 (0.59-1.26)	0.445	32 (19)	1.06 (0.68-1.65)	0.808	24 (14)	0.82 (0.5-1.33)	0.414	45 (27)	1.14 (0.77-1.68)	0.521
Pupils and students	188	65 (35)	1.38 (0.86-2.21)	0.185	43 (23)	0.84 (0.49-1.44)	0.521	36 (19)	1.06 (0.6-1.89)	0.838	52 (28)	1.04 (0.63-1.71)	0.888
Retired	669	186 (28)	0.88 (0.65-1.18)	0.398	84 (13)	0.93 (0.63-1.36)	0.699	101 (15)	0.92 (0.63-1.35)	0.671	139 (21)	0.95 (0.69-1.31)	0.756

Base: respondents who had taken antibiotics (n = 1919)

*sig.–statistically significant (in bold)

### Knowledge and opinions about antibiotics

The vast majority of the respondents (80%) recognized that antibiotics “kill” bacteria but at the same time 60% of respondents believed that antibiotics kill viruses as well. Gender, age, education, and employment factors were associated with knowledge about antibiotics (Table 3). Women (32%) were more likely than men (23) to give the correct answer that antibiotics kill bacteria but do not kill viruses (OR = 0.59, 95% CI = 0.51–0.68). Respondents with higher education level (46%) more frequently gave the appropriate answer compared with respondents with primary education (8.7%, OR = 0.11, 95% CI = 0.07–0.17) and trade school (13%, OR = 0.18, 95% CI = 0.14–0.23).

**Table 3:** Knowledge about antibiotics *(socio-demographic profile)*.

Characteristics	Waves 1–5 n	Antibiotics kill viruses (answer YES)			Antibiotics kill bacteria (answer YES)			Antibiotics kill bacteria, antibiotics do not kill viruses (answer YES)			Does imprudent use of antibiotics lead to antimicrobial resistance? (answer YES)			Waves 1–4 n	Antibiotics are effective against cold (answer YES)			Antibiotics are effective against flu (answer YES)		
		%	Adjusted ORs (95% CI)	sig.*	%	Adjusted ORs (95% CI)	sig.	%	Adjusted ORs (95% CI)	sig.	%	Adjusted ORs (95% CI)	sig.		%	Adjusted ORs (95%CI)	sig.	%	Adjusted ORs (95% CI)	sig.
**Total**	5004	60			80			28			86			4003	36			49		
**Sex**																				
Male	2243	66	1	-	78	1	-	23	1	-	88	1	-	1812	47	1	-	58	1	-
Female	2761	56	1.64 (1.44–1.86)	**<0.001**	81	0.83 (0.7–0.98)	**0.029**	32	0.59 (0.51–0.68)	**<0.001**	90	0.79 (0.66–0.96)	**0.016**	2191	29	2.16 (1.88–2.48)	**<0.001**	41	2.2 (1.91–2.54)	**<0.001**
**Age groups**																				
18–24	513	69	1	-	78	1	-	21	1	-	87	1	-	428	44	1	-	78	1	-
25–34	887	62	1.03 (0.75–1.41)	0.858	81	1.39 (0.96–2.03)	0.081	29	1.19 (0.84–1.69)	0.332	90	1.13 (0.73–1.77)	0.580	730	37	0.89 (0.64–1.23)	0.484	60	0.5 (0.35–0.72)	**<0.001**
35–44	812	57	0.71 (0.51–0.98)	**0.035**	83	1.69 (1.14–2.5)	**0.009**	34	1.96 (1.37–2.80)	**<0.001**	93	1.97 (1.21–3.20)	**0.006**	654	35	0.7 (0.5–0.98)	**0.039**	44	0.22 (0.15–0.33)	**<0.001**
45–59	1508	60	0.73 (0.53–1)	**0.048**	80	1.62 (1.12–2.34)	**0.010**	29	1.84 (1.30–2.61)	**0.001**	88	1.11 (0.72–1.70)	0.646	1210	34	0.64 (0.47–0.89)	**0.007**	43	0.2 (0.14–0.29)	**<0.001**
≥60	1284	59	0.67 (0.47–0.94)	**0.020**	77	1.45 (0.96–2.18)	0.075	26	1.80 (1.23–2.64)	0.002	86	0.93 (0.58–1.50)	0.779	981	34	0.65 (0.46–0.93)	**0.019**	38	0.19 (0.13–0.28)	**<0.001**
**Education**																				
University education	1442	45	1	-	81	1	-	46	1	-	94	1	-	1141	27	1	-	40	1	-
Primary education	400	77	6.17 (4.51–8.44)	**<0.001**	76	0.88 (0.63–1.23)	0.453	9	0.11 (0.07–0.17)	**<0.001**	77	0.25 (0.18–0.36)	**<0.001**	305	49	3.05 (2.3–4.05)	**<0.001**	61	3.53 (2.62–4.76)	**<0.001**
Trade school	960	74	4.04 (3.3–4.94)	**<0.001**	78	0.91 (0.71–1.17)	0.460	13	0.18 (0.14–0.23)	**<0.001**	84	0.38 (0.28–0.50)	**<0.001**	774	48	2.45 (1.99–3.01)	**<0.001**	59	2.56 (2.08–3.15)	**<0.001**
Secondary education	2195	62	2.14 (1.85–2.48)	**<0.001**	80	0.98 (0.8–1.2)	0.854	27	0.44 (0.38–0.52)	**<0.001**	90	0.60 (0.46–0.78)	**<0.001**	1776	34	1.45 (1.22–1.33)	**<0.001**	48	1.56 (1.32–1.84)	**<0.001**
**Employment**																				
Employed	2402	57	1	-	81	1	-	33	1	-	91	1	-	1924	35	1	-	50	1	-
Unemployed	473	68	1.31 (1.03–1.66)	**0.029**	80	0.97 (0.72–1.31)	0.854	22	0.81 (0.62–1.05)	0.107	85	0.72 (0.53–0.97)	**0.033**	383	40	1.32 (1.03–1.69)	0.221	58	1.32 (1.03–1.69)	**0.031**
Pupils and students	368	67	1 (0.71–1.4)	0.982	80	1.15 (0.76–1.74)	0.496	24	1.10 (0.76–1.59)	0.625	89	1.08 (0.66–1.77)	0.770	311	41	1.03 (0.7–1.53)	0.850	75	1.03 (0.7–1.53)	0.872
Retired	1761	61	1.29 (1.07–1.55)	**0.008**	78	0.92 (0.72–1.17)	0.474	24	0.73 (0.59–0.89)	0.002	87	0.98 (0.74–1.28)	0.858	1385	35	0.9 (0.74–1.1)	0.269	39	0.9 (0.74–1.1)	0.316

*sig.–statistically significant (in bold)

Males (66%,) more often than females (56%), declared that antibiotics were active against viruses (OR = 1.64, 95% CI = 1.44–1.86). The same opinion was also expressed by the younger respondents and those with lower education. The numbers of respondents who believed that antibiotics kill viruses decreased with age (Table 3).

In the first four waves of the survey respondents were also asked if antibiotics are effective in treating the common cold and flu. Many of the respondents believed that antibiotics are effective against flu (49%) and colds (36%). A greater number of males than females believed that antibiotics are effective against the cold (OR = 2.16, 95% CI = 1.88–2.48) and flu (OR = 2.2, 95% CI = 1.91–2.54). This opinion was more likely expressed by younger respondents between the age of 18–24 than older ones (e.g. flu respondents ≥ 60 OR = 0.19, 95% CI = 0.13–0.28) and with lower education compared to those with higher level (e.g. flu OR = 3.53, 95% CI = 2.62–4.76).

In the five waves of the study the respondents were asked if it was true or false that imprudent use of antibiotics leads to antimicrobial resistance. A large majority (86%) of those polled gave the correct answer. The distribution of answers was almost exactly the same in every of the five waves of the study (Table 3).

### Attitude toward antibiotic use

Approximately 41% of the respondents expected a prescription for an antibiotic against flu but less often against sore throat (23%), cold (19%), toothache (15%) and cough (10%). Males were more likely than females to expect an antibiotic prescription for flu (47% and 36%, OR = 1.54, 95% CI = 1.36–1.74)) and common cold (21% and 17%, OR = 1.42, 95% CI = 1.23–1.66)) and a similar opinion about flu was shared by younger respondents aged 18–24 (69%) and 25–34 (50%) as opposed to those aged 60 and over (31%)—(Table 4). Respondents with lower education more often expect prescription of antibiotics for colds (OR = 3.45, 95% CI = 2.61–4.56) and flu (OR = 2.2, 95%CI = 1.72–2.81).

**Table 4:** Attitudes about antibiotics and expected prescription of an antibiotic *(socio-demographic profile)*.

Characteristics	Wave 5 (n)	Current use of antibiotics should be restricted in order to keep them effective in the future (%)	Antibiotics should be prescribed and taken “just in case”, with the hope that other equally effective drugs will be developed (%)	Adjusted ORs (95% CI)	sig.*	Waves 1–5 (n)	Expected prescription of antibiotic for flu (%)	Adjusted ORs (95% CI)	Sig.	Expected prescription of antibiotic for cold (%)	Adjusted ORs (95% CI)	sig.
**Total**	1001	82	15			5004	41			19		
**Sex**												
Male	431	79	18	1	-	2243	47	1	-	21	1	-
Female	570	84	14	1.45 (1.01–2.09)	**0.044**	2761	36	1.54 (1.36–1.74)	**<0.001**	17	1.42 (1.23-1-66)	**<0.001**
**Age groups**												
18–24	85	75	24	1	-	513	69	1	-	17	1	-
25–34	157	89	10	0.33 (0.14–0.83)	**0.018**	887	50	0.54 (0.41–0.73)	**<0.001**	16	1.35 (0.92–1.97)	0.123
35–44	158	86	13	0.45 (0.19–1.08)	0.074	812	35	0.26 (0.19–0.36)	**<0.001**	16	1.17 (0.79–1.73)	0.431
45–59	298	79	19	0.63 (0.28–1.41)	0.260	1508	37	0.28 (0.21–0.38)	**<0.001**	20	1.35 (0.93–1.96)	0.110
≥60	303	82	14	0.30 (0.12–0.72)	**0.007**	1284	31	0.23 (0.16–0.31)	**<0.001**	21	1.5 (1.01–2.25)	**0.047**
**Education**												
University education	301	85	14	1	-	1442	34	1	-	11	1	-
Primary education	95	66	25	1.96 (1.07–3.62)	**0.031**	400	49	2.2 (1.72–2.81)	**<0.001**	32	3.45 (2.61–4.56)	**<0.001**
Trade school	186	82	16	0.94 (0.55–1.61)	0.826	960	47	1.89 (1.58–2.26)	**<0.001**	26	2.58 (2.06–3.24)	**<0.001**
Secondary education	419	84	14	0.84 (0.53–1.31)	0.434	2195	41	1.38 (1.19–1.59)	**<0.001**	18	1.65 (1.35–2.02)	**<0.001**
**Employment**												
Employed	478	85	14	1	-	2402	41	1	-	16	1	-
Unemployed	90	84	11	0.68 (0.32–1.45)	0.316	473	46	1.06 (0.85–1.31)	0.611	24	1.37 (1.07–1.77)	**0.014**
Pupils and students	57	77	21	0.90 (0.34–2.37)	0.833	368	67	1.15 (0.83–1.58)	0.403	17	1.2 (0.8–1.82)	0.380
Retired	376	79	18	1.78 (1.09–2.91)	**0.021**	1761	33	0.92 (0.77–1.09)	0.346	21	1.11 (0.9–1.38)	0.319

*sig.–statistically significant (in bold)

The survey were asked how the respondents obtained the course of antibiotics they used. Almost all (90%) of the respondents said that they had obtained their last course of antibiotics from a physician’s prescription. Only 3% of respondents stated that they had acquired an antibiotic from a pharmacy without a prescription.

The majority of the respondents (79%) had taken the full dose of the antibiotic prescribed. The most common reason for stopping antibiotic treatment early was resolution of symptoms (47%). More males (54%) than females (39%) claimed to have stopped taking antibiotics due to a relief in symptoms (OR = 1.94, 95%CI = 1.27–2.97). Additionally, young people (18–24, 41%) including high school/university students (38%) were less likely than older respondents (15%) to take the full dose of the antibiotic prescribed (OR = 0.28, 95% CI = 0.17–0.45). In the fifth wave of the survey, the respondents were additionally asked if the current use of antibiotics should be restricted in order to keep them effective in the future, or if they should be prescribed and taken “just in case”, with the hope that other equally effective drugs will be developed. Most respondents thought antibiotic use should be limited so that they could still be used effectively in the future (82%). Only 15% of the respondents believed that antibiotics should be taken”just in case”, while 3% claimed they had no knowledge on the matter. The youngest group of respondents, 18–24 years (24%) and with lower level of education (25%, OR = 1.96, 95%CI = 1.07–3.62) were most likely to take antibiotics”just in case” (Table 4).

### Sources of information

In all five waves of the study only 10% of respondents indicated having searched for any information about antibiotics. The most commonly indicated sources of knowledge were: websites dedicated to health (57%), health magazines (36%), health encyclopedias (33%), information from physicians (32%) and family and friends (19%). Respondents older than 60 less frequently than in other age groups searched information on the Internet (OR = 0.16, 95%CI = 0.08–0.32). The respondents were asked to give their opinion regarding which sources of information about antibiotics are the most trustworthy. Physicians (90%), hospital staff (67%), pharmacists (57%) and nurses (30%) were considered to be the most trustworthy sources of information.

In the fifth wave an additional question was introduced, regarding the opinion of the respondents on physicians’ attitude to prescribing an antibiotic. The majority of the respondents trust physicians in this regard (66%). Less than half (46%) believed that physicians often prescribe antibiotics unnecessarily and 28% stated that physicians often refuse to prescribe an antibiotic in serious cases. One third of the respondents (33%) believed that it is possible to pressure physicians to prescribe antibiotics. There were major differences in opinions regarding doctors’ attitudes based on age and education. Most respondents aged over 60 years (72%) were more ready to trust the physicians’ suggestions regarding antibiotics than the younger ones (p<0.001). This view was most frequently expressed by respondents with lower education level (73%) than with higher (64%) p<0.001. The opinion that physicians often prescribe antibiotics unnecessarily was expressed least frequently among the respondents with higher education than primary education (OR = 0.41, 95%CI = 0.24–071).

### Knowledge of the EAAD campaign

The respondents were asked if they had come across any information about the proper use of antibiotics in the preceding 12 months. One third of the respondents (29%) declared that they received such information. More women than men (32% versus 25%) came across information on the proper use of antibiotics (OR = 0.72, 95%CI = 0.63–0.82). People with higher education (35%) came across news on this topic more often than the other groups—people with trade school (22%, OR = 5.11, 95%CI = 0.42–0.62)) and primary education (19%, OR = 0.39, 95% CI = 0.29–051).

Those respondents who said they received information in the last 12 months about antibiotics were asked to identify the source of this information. The majority of the respondents received information about antibiotics from EAAD campaigns press 31%, television 28%, leaflets 16%, Internet 15% and physicians 23%.

### Impact of the European Antibiotic Awareness Day campaigns on perception and behavior

Starting with the third wave of the survey (October 2010) an increased number of polled respondents declared their views had been affected by the information they received. After the last wave of the survey (November 2011) significant changes in attitude were observed among the respondents in comparison with the results of the first survey (October 2009)—Table 5:

- fewer of the respondents had used antibiotics to treat cold (30% in 2009 and 21% in 2011, p = 0.008), sore throats (24% in 2009 and 12% in 2011, p<0.001) and flu (16% in 2009 and 13% in 2011,p = 0.124)
- more respondents declared they had taken the full dose of antibiotic prescribed (75% in 2009 and 81% in 2011, p = 0.063)
- fewer respondents expected a prescription for antibiotics because of colds (19% in 2009 and 15% in 2011, p = 0.019) and flu (43% in 2009 and 32% in 2011, p<0.001)
- more respondents declared a change in behavior after receiving EAAD campaign information (38% in 2009 and 48% in 2011, p = 0.012)

**Table 5:** Impact of the EAAD campaigns on perception and behavior (Waves 1–5).

Management	Wave 1 October 2009%	Wave 2 December 2009% (change)	Wave 2 vs.1 sig.*	Wave 3 October 2010% (change)	Wave 3 vs.1 sig.	Wave 4 December 2010% (change)	Wave 4 vs.1 sig.	Wave 5 December 2011% (change)	Wave 5 vs.1 sig.
**The reason for last taking antibiotic:**									
Cold	30	33 (↑3)	0.35	38 (↑8)	**0.021**	28 (↓2)	0.654	21 (↓9)	**0.008**
Sore throat	24	25 (↑1)	0.721	29 (↑5)	0.112	27 (↑3)	0.307	12 (↓12)	**<0.001**
Flu	16	18 (↑2)	0.465	18 (↑2)	0.658	14 (↓2)	0.341	13 (↓3)	0.124
**Expected a prescription of antibiotic because of:**									
Cold	19	18 (↓1)	0.801	22 (↑3)	0.06	20 (↑1)	0.569	15 (↓4)	**0.019**
Flu	43	41 (↓2)	0.345	46 (↑3)	0.143	41 (↓2)	0.319	32 (↓11)	**<0.001**
**Taking the full dose of antibiotic prescribed**	75	80 (↑5)	0.074	79 (↑4)	0.255	78 (↑3)	0.511	81 (↑6)	0.063
**Change in behavior after receiving EAAD campaigns information (YES):**	38	39 (↑1)	0.662	52 (↑14)	**0.002**	47 (↑9)	**0.014**	48 (↑10)	**0.012**
I have limited the use of antibiotics	27	43 (↑16)	**0.011**	38 (↑11)	0.073	42 (↑15)	**0.012**	43 (↑16)	**0.009**
I have become more cautious regarding the use of antibiotics	4	24 (↑20)	**<0.001**	19 (↑15)	**<0.001**	20 (↑16)	**<0.001**	16 (↑12)	**0.001**

* sig.–statistically significant (in bold), significance obtained using Chi^2^-test

It was observed that more respondents who came across the campaign indicated the following responses (Table 5):

- I have limited the use of antibiotics (27% in 2009 and 43% in 2011, p = 0.009)
- I have become more cautious regarding the use of antibiotics (4% in 2009 and 16% in 2011, p = 0.001)

Differences were observed between those with and without knowledge of the EAAD campaign with respect to stated reasons for antibiotics and expectations regarding their prescription for certain infections (Table 6). Those who had come across the EAAD campaign presented better knowledge than those who were not aware of the campaign, they less frequently expected a prescription of antibiotic for cold and flu.

**Table 6:** Impact of the obtained information on perception and behavior.

	n	Antibiotics kill viruses	Expected a prescription of antibiotic		n	Antibiotics are effective against cold	Antibiotics are effective against flu
		Waves 1–5 (answer YES) %	Waves 1–5 (answer YES) Cold %	Waves 1–5 (answer YES) Flu %		Waves 1–4 (answer YES) %	Waves 1–4 (answer YES) %
**Respondents who in the preceding 12 months had come across information on the correct use of antibiotics**	1442	49	13	34	1150	27	38
**Respondents who in the preceding 12 months had not received information on the correct use of antibiotics**	3562	65	21	44	2842	40	53
**P-value (Chi^2^-Test)**		**<0.001**	**<0.001**	**<0.001**		**<0.001**	**<0.001**

## Discussion

One of the most important steps in fighting antimicrobial resistance is augmenting the knowledge of the general public. Educational campaigns for the public play a vital role in this area [6]. Creating a good campaign requires actual knowledge of the target population and continual assessment of the campaign’s effectiveness. This is the first such study to be conducted in Poland which so broadly analyzed the awareness and attitude of the general public toward antibiotics and antibiotic resistance both before and after educational campaigns (organized within the frame of EAAD).

Our study results indicate that about 40% of respondents took antibiotics during the year preceding the poll. The main reasons were common cold, sore throat, flu, and cough. These findings correlated well with the results of studies from many other countries as well as a previous Polish study and that of Eurobarometer’2016 regarding Poland [7, 8].

Although the incidence of declared antibiotic use remained the same throughout our study, some positive changes in expectations for the prescription of antibiotics for flu, cold and sore throat, cough were seen.

European as well as international studies on the social knowledge of antibiotic use and antimicrobial resistance indicate a widespread ignorance regarding the ineffectiveness of antibiotic treatment for viral infections along with the knowledge of the difference between bacteria and viruses [9–20]. These studies highlight a lack of understanding of two correlated facts. First, that antibiotics do not kill viruses and second that antibiotics cannot be used to effectively treat viral infections, such as the common cold and flu. Studies from Sweden and Kuwait confirm a confusion among the public regarding whether antibiotics are effective against bacteria or viruses [9, 19]. This problem is stressed also in a publication from the USA in which 48% of respondents agreed with items that assess the "germs are germs" hypothesis, whereas 76% agreed with items that assess " why not take a risk" hypothesis [21]. The present study confirms these observations, showing that a high percentage of respondents not only did not understand the difference between viral and bacterial infections, but also did not understand the indications for antibiotic treatment.

Insufficient social awareness of antibiotic resistance leads to inaccurate expectations for antibiotic prescription. Our study suggested that Polish respondents would often expect antibiotic treatment in the case of typical viral infections, such as the common cold or flu. For this reason the EAAD campaign emphasized the fact that antibiotics are effective only against bacteria, highlighting this by the campaign slogan “Cold? Flu? Take care without antibiotics! Antibiotics don’t kill viruses and don’t treat viral illnesses”.

Social ignorance was also widely visible in Eurobarometer surveys, where large numbers of Europeans were unaware that antibiotics are ineffective against the cold and flu (2013–41%, 2016–44%) [8, 22].

Various studies carried out worldwide, along with our own study, indicated that age and education level as well as gender are major factors affecting attitudes towards antibiotics [23–28]. Our study also demonstrated that young adults were the most likely to believe in the effectiveness of antibiotic treatment against viruses. Educational level was also found to have an effect as people with lower education levels were found to have lower levels of awareness that antibiotics are ineffective against viruses.

The world literature indicates that the percentage of bacterial resistance to antibiotics is correlated with the intensity of usage of these drugs [29]. Certainly, then, self-medication plays an important role in driving resistance [30, 31]. Two types of behavior are known to contribute to this phenomenon: obtaining antibiotics from a pharmacy without a prescription or obtaining them from a non-medical source (e.g. left over from a previous course). In Poland, other European Union countries, and in North America an antibiotic may be obtained only by medical prescriptions. It seems, according to our own research, that the issue of self-medication in Poland is insignificant, since the vast majority of Poles used antibiotics prescribed by a doctor or dentist and only 3% bought such drugs at a pharmacy without a prescription, which is not in accordance with Polish law.

Similar results came from Eurobarometer surveys of both 2009 and 2013 on self-medication in which 98% Poles acquired antibiotics by prescription while EU countries’ average was 95% [22, 32]. Self-medication can also mean the use of antibiotics remaining from previous therapy, although recently published results of Eurobarometer 2016 survey for Poland showed that the number of respondents who obtained the antibiotics 'not from a medical practitioner' has significantly increased (9%).

In many countries of the world antibiotics can be purchased without medical prescriptions. This topic was the subject of intense focus in the national campaigns of Greece and Spain, hoping that public education would decrease antibiotic usage outside of a medical professional’s prescription [6]. Success in this field was noted in Australia, where a drop in self-medication with antibiotics for flu, colds and coughs from 10.8% in 1999 to 7.4% in 2004 was achieved as a result of educational campaigns [33].

Another important element of protection from development of AMR is the correct use of antibiotics. The importance of compliance following physician recommendations concerning antibiotic dosage, appropriate intervals, and duration of therapy has been widely described in the literature. As shown in our study, although the vast majority of respondents (79%) take a full dose of prescribed antibiotic, there is still a group of mostly young adults, who do not follow the prescribed regimen and discontinue treatment prematurely mainly due to relief of symptoms. Similar results were obtained in studies conducted in other countries, including Malaysia (71.1%) and Hong Kong (58%) [26, 34]. It is worth mentioning that in some countries a more responsible attitude toward antibiotic use is observed. An example is Sweden, where the population seems to be the most disciplined with only 4.5% of people admitting early discontinuation of therapy if they feel better [9].

In order to improve knowledge on antibiotics and accompanying risks of their misuse, access to proper information for the general public is much needed.

Research conducted among Greek parents showed that 90% of them received information on antibiotics from pediatricians [35]. Also, respondents from Italy (80.1%) indicated physicians as the main source of information on antibiotics [23]. In Eurobarometer respondents also are most likely to say that they received information about antibiotics from a doctor (2016–32%) [8]. In our study the opinion is shared by Poles as doctors, pharmacists and nurses were indicated as the most reliable sources of antibiotic information.

The role of the Internet as a promoter of health issues was confirmed by research done in the UK [36–38]. It has been shown that the websites on health can play a significant role in raising public awareness. In Eurobarometer2016 13% of respondents who had received information about not taking antibiotics unnecessarily saw it on the Internet or on online social media (PL in Eurobarometer'2016–19%) [8]. More than 30% of Poles have an account on at least one social network. This information should be taken into account when planning targeted educational activities with the use of new informational tools. Also in Poland the Facebook fan page National Program of Antibiotics Protection has been quite successful in distributing knowledge on antibiotics and antibiotic resistance, playing an important role in creating the right attitudes towards expectations for health professional prescription of antibiotics (http://www.antybiotyki.edu.pl/).

No sufficient change from education campaigns regarding the viral or bacterial nature of certain infections was reported from New Zealand (1998 and 2003), England and Scotland (2008, 2009) [20, 39]. On the other hand, antibiotic campaigns conducted in Belgium, England and France that used high impact tools (e.g. use of advertisements via radio, television, print media, billboards) and were repeated for several years resulted in improved antibiotic use and attitudes [40–43]. In Eurobarometer'2016 around a third (34%) of the respondents who said they had received information about antibiotics said that their views were changed by this information [8].

The results of EAAD campaigns conducted in Poland allow for slight optimism. The impact of our campaign on behavioral changes toward antibiotic use was seen; nearly half of the participants claimed a change in their opinions based upon the information obtained in this campaign. Also, a favorable trend was noted among Polish respondents participating in the Eurobarometer (2013,2016) surveys, which indicated an increase and sustainability of knowledge on the lack of antibiotic activity for viral infections and the ineffectiveness of their use in these situations [8, 22].

Several studies have shown that there is a growing concern expressed by the general public regarding increasing antibiotic resistance [3]. The Polish public fared well in Eurobarometer studies and answered correctly that the unnecessary use of antibiotics makes them become ineffective (85% in 2009, 2013 and 81% in 2016) [8, 22, 32]. This result was consistent with the opinion of Poles expressed in the present study.

## Conclusion

The survey has generated information about knowledge, attitudes, and behaviors regarding antibiotics among the general population of Poland. Inappropriate antibiotic use is still highly prevalent in Poland, although a positive trend was observed in behavioral change after educational campaigns.

The study results show the diversity of public awareness regarding antibiotics and antimicrobial resistance on the basis of socio-demographic factors and indicated that young people and those with lower level of education should be the main target of future educational campaigns. Hopefully the results of the study will stimulate innovative and highly specific actions to provide educational tools to the general public aimed at improving social knowledge regarding antibiotics and the consequences of their misuse. Additional didactic and systematic education campaigns regarding appropriate antibiotic use are needed and the Internet as an education tool should be enhanced.

## Appendix

### Detailed topics of the questionnaire

1. Use of antibiotics, including previous antibiotic exposure:
  - Antibiotic use during the past year
  - Reasons for taking antibiotics
  - Ways of obtaining antibiotics
2. Knowledge about antibiotics:
  - Do antibiotics kill viruses?
  - Are antibiotics effective against the common cold and flu?
  - Does imprudent use of antibiotics lead to antimicrobial resistance?
3. Attitudes and behaviors about antibiotics
  - Patient expectations of antibiotic prescription
  - Use of antibiotics without prescription
  - Reason for discontinuation of antibiotic therapy
  - Should the current use of antibiotics be restricted in order to keep them effective in the future?
4. Sources of information
  - Sources of knowledge on antibiotics
  - The most trustworthy sources of information
  - Public attitudes towards physicians prescribing an antibiotic
5. Knowledge and impact of the campaign on attitudes towards the use of antibiotics.
